# Circular Distribution Pattern of Plant Modulars and Endophagous Herbivory within Tree Crowns: The Impact of Roadside Light Conditions

**DOI:** 10.1673/031.013.14101

**Published:** 2013-12-30

**Authors:** Xiao-Hua Dai, Jia-Sheng Xu, Xing-Lu Ding

**Affiliations:** 1 School of Life and Environmental Sciences, GanNan Normal University, Ganzhou 341000, China; 2 National Navel-Orange Engineering Research Center, Ganzhou 341000, China

**Keywords:** circular statistics, Fagaceae, leaf galls, leaf mines, sun vs. shade responses

## Abstract

The circular distributions of plant modulars (branches, leaves) and endophagous herbivory (mines, galls) were investigated within the crowns of four dominant Fagaceae trees in a subtropical evergreen broadleaf forest at Jiulianshan National Nature Reserve, Jiangxi, China. The hypothesis is that more plant modulars and more endophagous herbivory should occur in the crown area perpendicular to the roads. Circular statistical techniques were used to verify new patterns of the impact of roads on plants and insects. The results confirmed that the roadside light environments had larger impacts on the circular distribution patterns of plant modulars than those of leaf herbivores. For herbivores, the impact of light was larger on mine distribution than on gall distribution. The branches of all four tree species were concentrated in the direction perpendicular to the roads. In the preferred direction, branches were longer and higher. More leaves, more mines, and more galls were found surrounding the preferred branch direction. In general, leaf miners and leaf gallers preferred leaves in the sun over those in the shade; however, leaf gallers had a lower degree of preference for sun than leaf miners. Different endphagous insects also showed clear interspecific differences in sun/shade leaf selection.

## Abstract (ch)

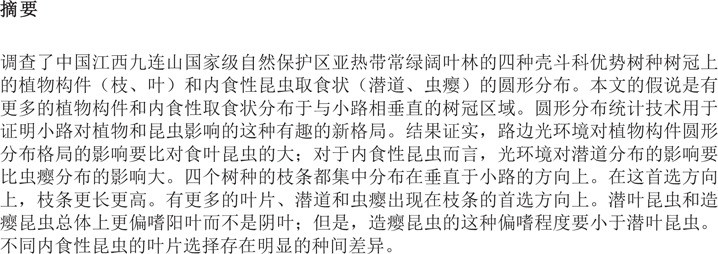

## Introduction

Shade lessens the number of branches and leaves (Potter 1992; Huber and Stuefer 1997; Bartlett and Remphrey 1998; Lin et al. 2007; Lusk et al. 2008). Leaves in the sun and shade have different physical and chemical characteristics such as leaf size, leaf shape, leaf color, leaf trichome, nutrient content, secondary metabolites, and leaf temperature (Faeth et al. 1981; Rotheray 1987; Collinge and Louda 1988; Basset 1991; Faeth 1991a; Potter 1992; Moller 1995; Connor 2006; Pincebourde et al. 2007). The sun vs. shade distribution of natural enemies and endophytic fungi might affect the leaf miners and plant gallers (Fernandes and Price 1992; Wilson and Faeth 2001; Gange et al. 2002; Gange et al. 2007; Horvath and Benedek 2009). Thus, light will act directly or indirectly on the oviposition selection of leaf miners and leaf gallers (Connor 2006; Dai et al. 2011).

Different miners and gallers show different preference for leaves in the sun or shade on an individual plant. Some leaf miners, such as *Phyllonorycter maestingella, Stigmella hemargyrella, S. tityrella, Cameraria* spp., and *Fenusa absens,* occur more on shaded leaves (Nielsen and Ejlersen 1977; Basset 1991; Faeth 1991a; Faeth 1991b; Smith and Altenhofer 2011); *Eriocraniella* spp., *Neurobathra strigifinitella, Dyseriocrania* spp., *Acrocercops* spp., *Bucculatrix cerina, Rhynchaenus fagi, Stilbosis quadricustatella,* and *Phytomyza ilicicola* are found more on leaves in the sun (Nielsen and Ejlersen 1977; Faeth et al. 1981; Kimmerer and Potter 1987; Simberloff and Stilling 1987; Horvath and Benedek 2009); *Cameraria hamadryadella* and *Eriocrania* spp. prefer equally sun and shade leaves (Brown et al. 1997; Riihimaki et al. 2003; Connor 2006); and *Br achy s tessellatus* and *Cameraria ohridella* change their choices on leaves in the sun or shade in different seasons (Turnbow and Franklin 1981; Horvath and Benedek 2009). For plant gallers, some prefer leaves in the sun while others show no preference between leaves in the sun and leaves in the shade (Nielsen and Ejlersen 1977; Basset 1991; Leite et al. 2009).

The studies mentioned above divided a plant crown into two parts, those in the sun and those in the shade. However, branches, leaves, and herbivory in different crown microhabitat area will experience a gradient variation of light levels. If the crown is projected as a circle on a plane, then circular statistical techniques can be adopted to study directional patterns such as crown displacement (Ackerly and Bazzaz 1995; Aradottir et al. 1997; Rouvinen and Kuuluvainen 1997; Muth and Bazzaz 2003; Tarara et al. 2005; Getzin and Wiegand 2007; Schröter et al. 2011), branching and leafing characteristics (Neufeld et al. 1988; Hollinger 1989; Doruska and Burkhart 1995; Gielen et al. 2002; Trincado 2006), and herbivory (Alonso 1997; Bonebrake et al. 2010).

After preliminary investigation, the number of branches, number of leaves, number of leaf mines per leaf, and number of leaf galls per leaf seemed to be different in different tree crown areas. Moreover, plant modulars and leaf herbivores were concentrated in the crown area closer to the roads. In order to show the sophisticated impacts of roadside light environments on plants and insects, circular statistics were adopted to quantify and verify distribution patterns around tree crowns. The following two questions were addressed: (1) Is the density of leaf miners and leaf gallers higher in some specific tree crown areas? (2) Are there interspecific differences in circular distribution patterns among leaf miner species and leaf galler species?

## Materials and Methods

### Study site

The 134-km^2^ Jiulianshan National Natural Reserve is located in the south of Jiangxi Province, China (24° 29′ 18″–24° 38′ 55″ N, 114° 22′ 50″–114° 31′ 32″ E). It is a mountainous area with an altitude range from 280 to 1,434 m a.s.l. The climate is subtropical, with a mean annual precipitation of 2,156 mm that occurs mainly in the wet season, from February to September. The mean monthly temperature varies from 6.8° C (January) to 24.4° C (July). Major vegetation types include subtropical evergreen broadleaf forest, low hill coniferous forest, bamboo forest, montane dwarf forest, and montane grassland (Liu et al. 2002).

### Data collection

**Figure 1.**
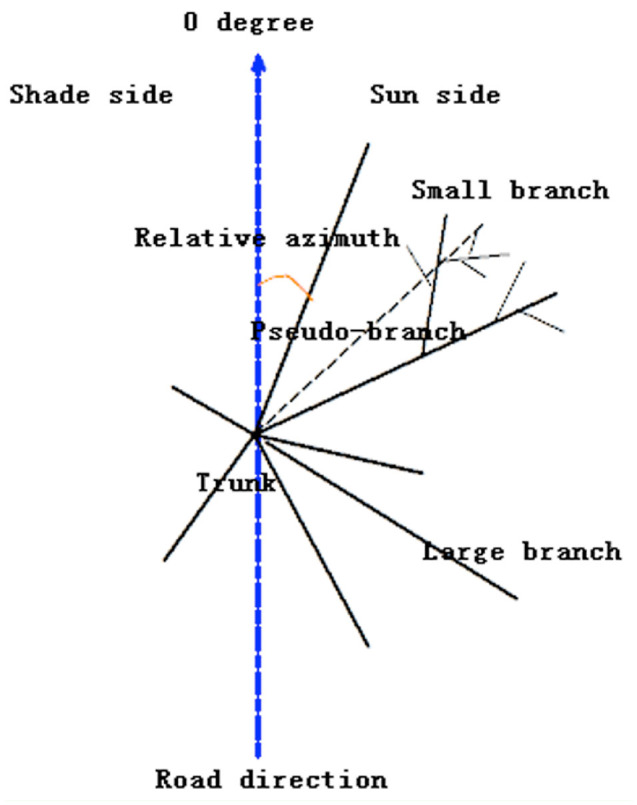
Sampling design of circular distribution data. Each sampled tree was located beside a small forest road. Road direction (thick dashed line) is from lowermost (as 180°) to uppermost (as 0°). Thus the tree crown was divided into two parts: sun side and shade side. Leaves were collected from the outermost 0.5 m section of each large branch (thick solid line). For small branches longer than 0.5 m (thin solid line) that were not located from the above section, a pseudo-branch (thin dashed line) was built in order to collect as many leaves as possible. The direction of a pseudo-branch was pointed to the center of the small branch bundle. Relative azimuth α is the angle between the large branch (and pseudo-branch) and the road, with clockwise as the positive sense of rotation. High quality figures are available online.

In July 2011, leaf herbivory was examined within the crowns of half-shaded understory trees beside small forest roads (Figure 1). Three *Castanopsis carlesii* (Hemsl.) Hayata (Fagales: Fagaceae) individuals with 50 branches, four *C. fabri* Hance with 90 branches, five *C. fargesii* Franch. with 84 branches, and six *Cyclobalanopsis glauca* (Thunb.) Oerst. with 81 branches were sampled. Leaves from the outermost 0.5 m sections of the branches (including large branches and pseudo-branches) were collected and counted for the number of leaves, leaf mines, and leaf galls. The types of leaf mines and leaf galls were decided according to their shape, position, and color (Appendix 1, 2) (Hering 1957; Csóka 1997, 2003; Redfern 2011). The fresh leaf miners were collected from time to time and reared in the laboratory, and photos of leaf miners were sent to taxonomists for identification. The density of one leaf mine type or one leaf gall type was measured as the number of leaf mines or leaf galls per leaf in each branch. Road directions were pointing from 180° to 0°. The whole crown can be divided into two sides, sun side (0–180°) and shade side (180–360°). Relative azimuths α of each large branch to the road direction were also measured. Branch length was measured from the base of branch to the farthest leaf. Branch height was the height of branch base. The base of one pseudo-branch was put at the base of the large branch where the longer small branch belonged (Figure 1).

### Statistical analysis

**Figure 2.**
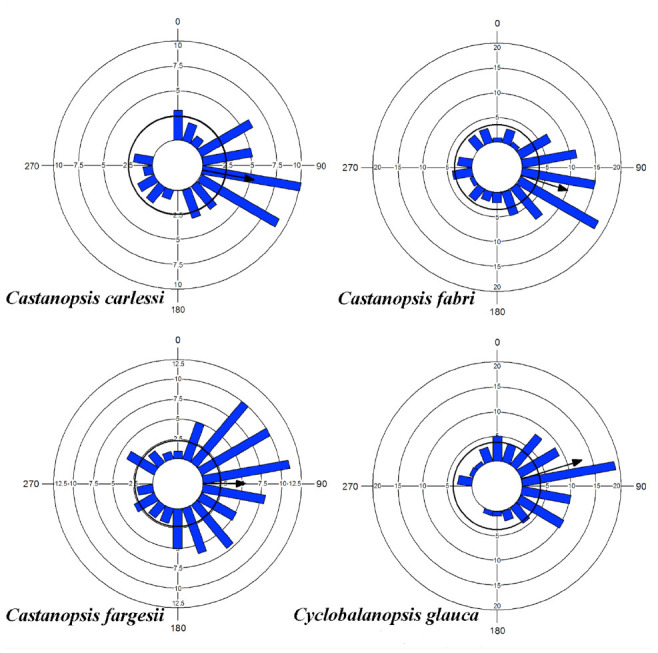
Circular histograms of branches' relative azimuths α within the crowns of four trees. Circular bars indicate the number of observations within each class range and have been centered on 0°. Mean relative azimuth μ is depicted as an arrow, and the arrow length represents the value of *r*. A solid-line circle was plotted across the bars on each histogram to show the level of the Rayleigh critical value. See text and Figure 1 for more details. High quality figures are available online.

Data from different individuals of the same tree species were pooled together. Relative azimuths α are angular data. Such angular measurements could be treated as points on one circle. Then, circular statistics were used to compute the mean vector. The mean vector has two properties: mean angle μ and its length *r*. *r* falls in the interval [0, 1], *r* is close to 1 for the data highly concentrated around one direction, and close to 0 for widely dispersed data. Rayleigh's uniformity test was performed to assess the significance of *r* (Zar 1999; Jammalamadaka and Sengupta 2001; Dai et al. 2007). V-test was used to test whether circular data *a* have a mean of 90°. A circular histogram was plotted for α, and a circle was drawn on the histogram to show the level of the Rayleigh critical value (*p* = 0.05 here). If the *r* vector extends beyond the circle, then the Rayleigh test is significant (Figure 2).

For the circular distribution of plant modulars and leaf herbivores, vector pairs were defined to consist of relative azimuths α as a circular variable and one other parameter as a weight variable. The latter could be branch length, branch height, number of leaves per branch, number of leaf mines per leaf, number of leaf galls per leaf, etc. Weighted mean vector (WMV) and its length *r* (scaled 0–1) were obtained. Moore's modified Rayleigh test was used to test the significance of *r* here (Zar 1999).

All statistical analyses were conducted in Oriana version 4.00 (Kovach Computing Services, www.kovcomp.co.uk/oriana).

## Results

### Circular distribution of branches and leaves

Mean relative azimuth μ of branch direction was 100.5° for *C. carlesii*, 107.7° for *C. fabri,* 89.3° for *C. fargesii,* and 72.7° for *C. glauca* (Figure 2). The corresponding *r* was 0.528, 0.491, 0.423, and 0.637 respectively. Relative azimuths a were not evenly distributed in all directions (Rayleigh tests, *Z* = 13.941, n = 50, *p* < 0.01; *Z* = 21.681, n = 90, *p* < 0.01; *Z* = 15.038, n = 84, *p* < 0.01; and *Z* = 32.829, n = 81, *p* < 0.01 for *C. carlesii, C. fabri, C. fargesii,* and *C. glauca* respectively). Moreover, a concentrated around 90° (V-tests, *V* = 0.519, n = 50*, p* < 0.01; *V* = 0.468, n = *90, p* < 0.01; *V* = 0.423, n = 84*, p* < 0.01; and *V* = 0.608, n = 81, *p* < 0.01 for *C. carlesii, C. fabri, C. fargesii,* and *C. glauca* respectively).

The WMV of relative azimuths weighted by branch length, by branch height, and by number of leaves per branch were all between 90 ± 30° for four tree species. Moore's modified Rayleigh test showed that all WMVs were significantly concentrated (*p* < 0.01) and all *r* values were larger than 0.167 (Table 1).

### Circular distribution of leaf miners and leaf gallers

The composition of leaf miners and leaf gallers was different between the three *Castanopsis* trees and the *Cyclobalanopsis* tree (Table 2).

The WMVs of relative azimuths weighted by the density of most leaf mine types (FLMO1, FLM05, FLM07, FLM09) and by the density of all leaf mines were all between 90 ± 30° for all four tree species. The WMVs of FLM02 and FLM03 were outside 90 ± 30° but within the range of 0 to 180° (sun side). The WMV of FLM04 was within the sun side but not always between 90 ± 30°. Moore's modified Rayleigh test indicated that all WMVs were significantly concentrated (*p* < 0.01) while all *r* values were between 0.018 and 0.116 (Table 2).

**Table 1.**
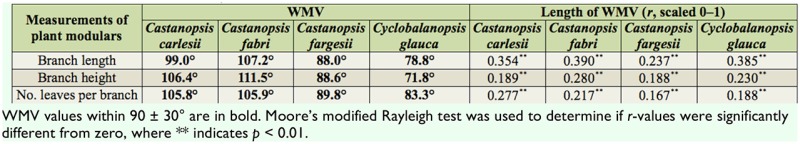
Circular distribution of plant modulars within the crowns of four trees.

**Table 2.**
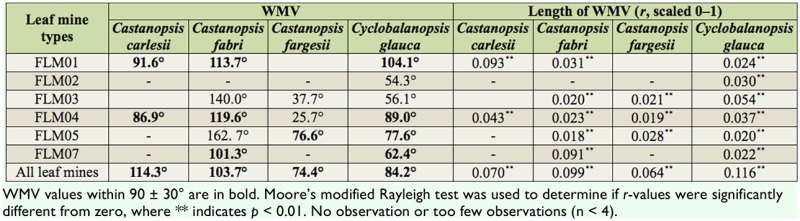
Circular distribution of leaf mine density (number per leaf) within the crowns of four trees.

**Table 3.**
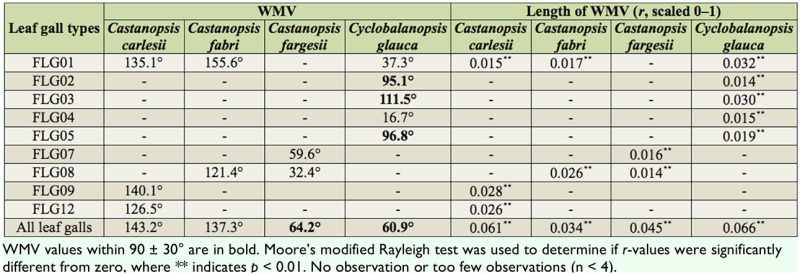
Circular distribution of leaf gall density (No. per leaf) within the crowns of four trees.

The WMVs of relative azimuths weighted by the density of each leaf gall type were outside 90 ± 30° but within 0–180° for three *castanopsis* species. The WMs of relative azimuths weighted by leaf gall types were FLM05, FLM07, FLM09) and by the density of all leaf mines were all between 90 ± 30° for all four tree species. The WMVs of FLM02 and FLM03 were outside 90 ± 30° but within the range of 0 to 180° (sun side). The WMV of FLM04 was within the sun side but not always between 90 ± 30°. Moore's modified Rayleigh test indicated that all WMVs were significantly concentrated (*p* < 0.01) while all *r* values were between 0.018 and 0.116 (Table 2).

The WMVs of relative azimuths weighted by the density of each leaf gall type were outside 90 ± 30° but within 0–180° for three *Castanopsis* species. The WMVs of relative azimuths weighted by leaf gall types were within 0–180° (sun side) but not always between 90 ± 30° for *C. glauca.* For the leaf gall types, the WMVs of FLG01, FLG04, FLG07, FLG08, FLG09, and FLG12 were outside 90 ± 30°, while the WMVs of FLG02, FLG03, and FLG05 were between 90 ± 30°. For *C. glauca*, The WMV variations were very evident. The WMVs of realtive azimuths weighted by the density of all leaf galls in four trees were generally away from 90°. Moore's modified Rayleigh test indicated that all WMVs were significantly concentrated (*p* < 0.01) while all *r*-values were between 0.014 and 0.061 (Table 3).

## Discussion

The length of WMVs (*r*) could be ordered as follows: *r* (branch direction) > *r* (branch length) > *r* (branch height) or *r* (No. leaves per branch) > *r* (mine density) > *r* (gall density) (Tables 1–3). It seems that light environment had a larger impact on the circular distribution of plant modulars than on that of leaf herbivores. For herbivores, light impacts were larger on mine distribution than on gall distribution. The branches of all four tree species were concentrated in the direction perpendicular to the roads (Figure 2). In the preferred direction, branches were longer and higher. There were also more leaves, mines, and galls around the preferred direction of branches (Tables 1–3).

Generally, leaf miners and leaf gallers preferred sun leaves rather than shade leaves. However, leaf gallers had a lower degree of preference to sun than leaf miners. The sun vs. shade distribution patterns were consistent with some studies (Faeth et al. 1981; Hartman 1984; Fernandes and Price 1992), but not with others (Basset 1991; Dudt and Shure 1994; Fernandes et al. 2004). Generally, leaf mines were found on the upper side of leaves while leaf galls were on the lower side of leaves, which might explain their differences in sunshade pattern.

Different endophagous insects showed clear interspecific differences in leaf selection. For example, the mines of Rhynchaeninae weevils (FLM07) were mostly found in leaves fully exposed to the sun, which is consistent with other leaf-mining beetles (Nielsen and Ejlersen 1977; Turnbow and Franklin 1981; Waddell and Mousseau 1996); the mines of *Stigmella* spp. Goze (Lepidoptera: Nepticulidae) (FLM01 and FLM03) were also more prevalent in leaves in the sun, which is contrary to the previous observations (Nielsen and Ejlersen 1977; van Nieukerken 2006). The mines of *Phyllonorycter* spp (FLM02) were not concentrated in fully-sun leaves, which is the same as other *Phyllonorycter* (Nielsen and Ejlersen 1977). The mines of *Acrocercops* spp (FLM04) were generally more in the sun side, which is same as other *Acrocercops* on Fagaceae trees (Faeth et al. 1981). The mines of *Tischeria* spp (FLM05) also preferred leaves in the sun, which is different than other *Tischeria* (Connor 2006).

Light intensity, light quality, and light time are different in different crown areas. The sun vs. shade leaves in our study were identified according to the road, not to the absolute south or north compass direction (Basset 1991). From this view, more plant modulars and a higher density of herbivorous insects occurred in the crown area perpendicular to the roads, indicating that high light intensity and long light hours are beneficial factors for these organisms. Compared to the traditional sun-shade analysis, circular analysis could provide a more detailed picture of light impact on plants and insects. Our next study is to measure circular distribution of light parameters and leaf chemical content around tree crowns and to connect the two investigations to discover leaf selection mechanisms of leaf-mining insects. Moreover, the concentration of leaf-feeding insects in specific areas might help control them precisely, for example, to release natural enemies or to apply pesticides only in the target area.

## Figures and Tables

**Appendix 1.**
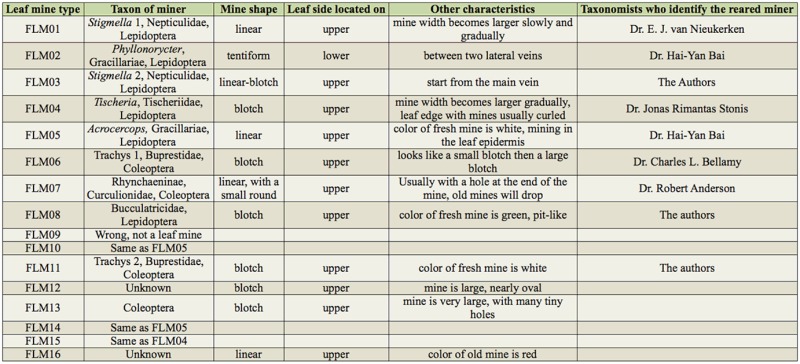
The identification of leaf mine types on four Fagaceae trees.

**Appendix 2.**
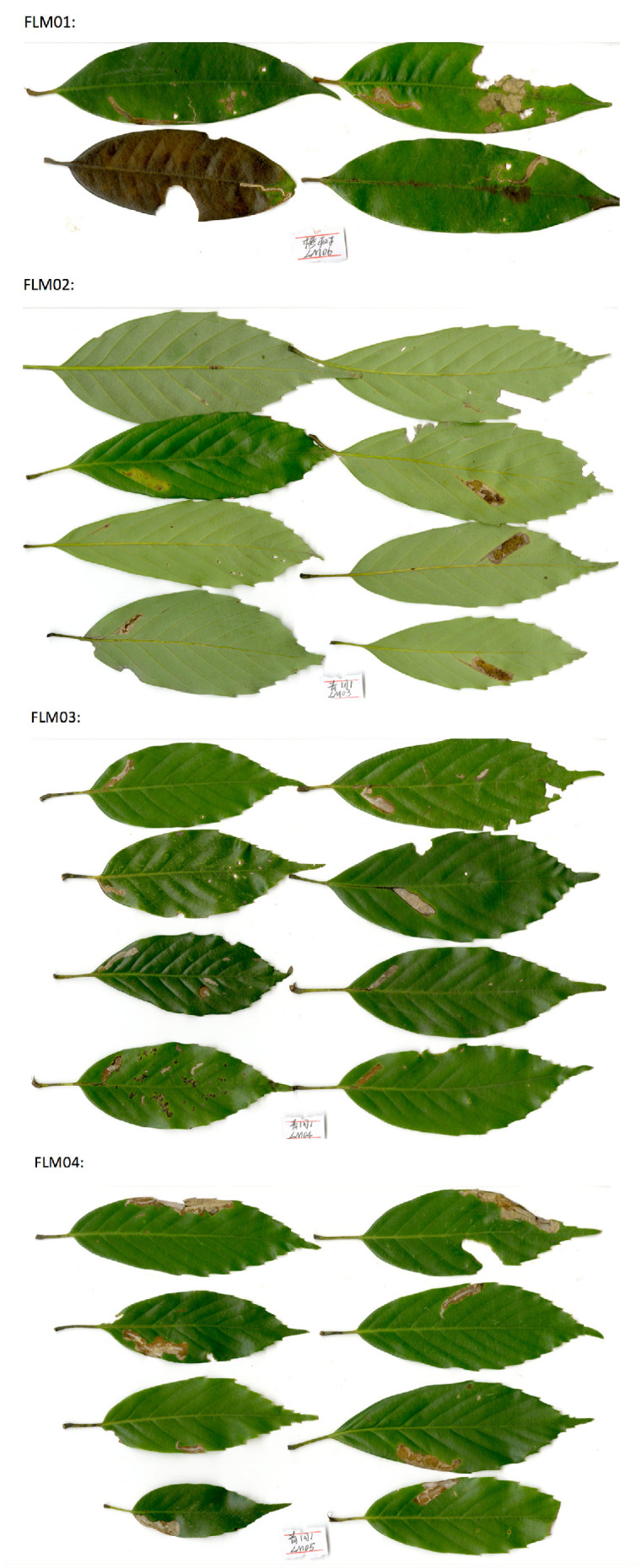
The figures of common leaf mine types on four Fagaceae trees.

**Appendix 3.**
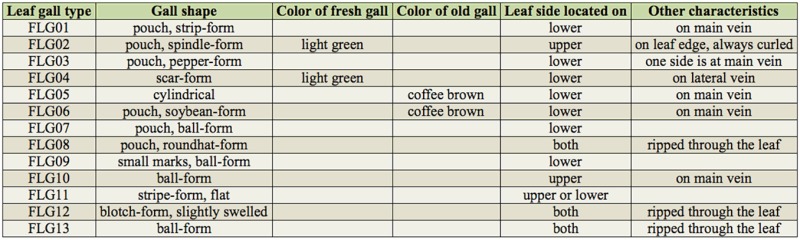
The identification of leaf gall types on four Fagaceae trees.

**Appendix 4.**
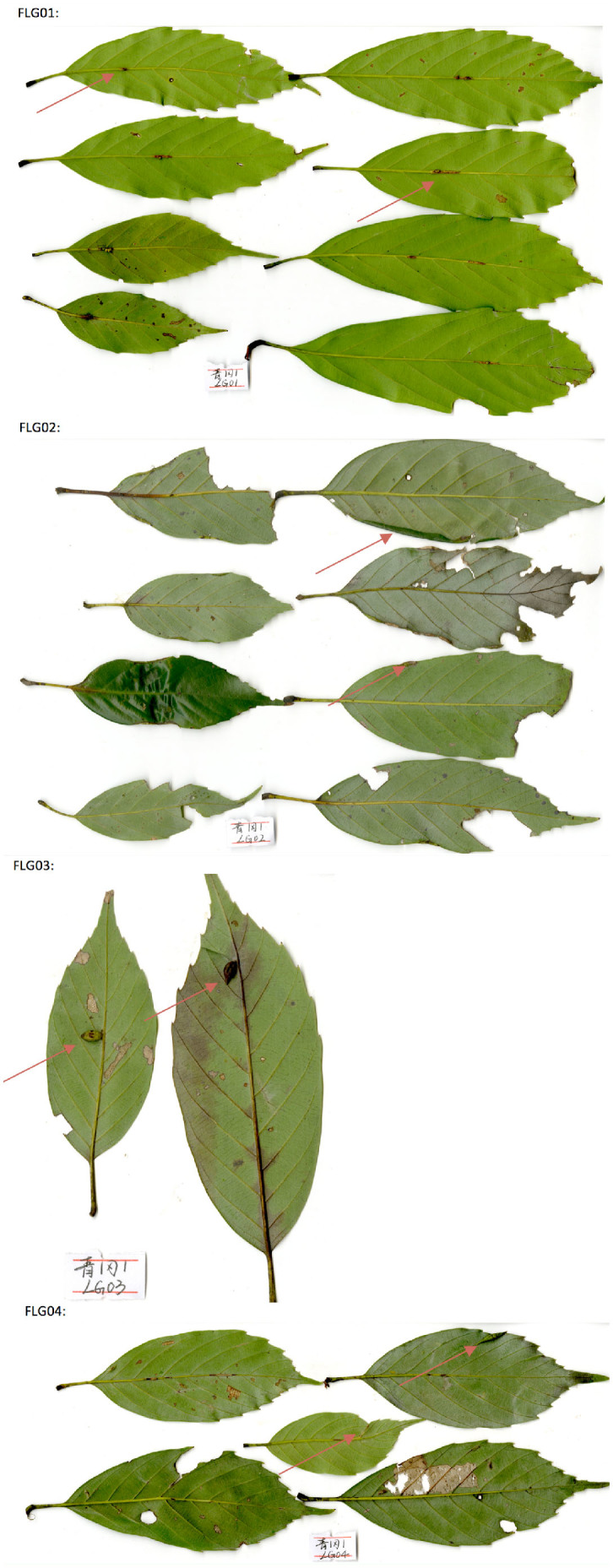
The identification of leaf gall types on four Fagaceae trees.

## Abbreviations

WMV,: weighted mean vector
